# BACE2 suppression in mice aggravates the adverse metabolic consequences of an obesogenic diet

**DOI:** 10.1016/j.molmet.2021.101251

**Published:** 2021-05-17

**Authors:** Daniela Díaz-Catalán, Gema Alcarraz-Vizán, Carlos Castaño, Sara de Pablo, Júlia Rodríguez-Comas, Antonio Fernández-Pérez, Mario Vallejo, Sara Ramírez, Marc Claret, Marcelina Parrizas, Anna Novials, Joan-Marc Servitja

**Affiliations:** 1 Pathogenesis and Prevention of Diabetes Group, Institut d'Investigacions Biomèdiques August Pi i Sunyer (IDIBAPS), Barcelona, Spain; 2 Centro de Investigación Biomédica en Red de Diabetes y Enfermedades Metabólicas Asociadas (CIBERDEM), Spain; 3 Instituto de Investigaciones Biomédicas Alberto Sols, Consejo Superior de Investigaciones Científicas (CSIC)/Universidad Autónoma de Madrid, Madrid, Spain; 4 Neuronal Control of Metabolism (NeuCoMe) Laboratory, Institut d'Investigacions Biomèdiques August Pi i Sunyer (IDIBAPS), Barcelona, Spain; 5 School of Medicine, Universitat de Barcelona, Barcelona, Spain

**Keywords:** BACE2, High-fat diet, β-cell proliferation, Insulin secretion, Insulin resistance, Leptin, AD, Alzheimer's disease, CD, Chow diet, BACE1, β-site APP-cleaving enzyme 1, BACE2, β-site APP-cleaving enzyme 2, BKO, BACE2 knock-out, GSIS, Glucose stimulated insulin secretion, HFD, High-fat diet, hIAPP, human islet amyloid polypeptide, TMEM27, Transmembrane protein 27, T2D, Type 2 diabetes, WT, Wild type

## Abstract

**Objective:**

Pancreatic β-cell dysfunction is a central feature in the pathogenesis of type 2 diabetes (T2D). Accumulating evidence indicates that β-site APP-cleaving enzyme 2 (BACE2) inhibition exerts a beneficial effect on β-cells in different models of T2D. Thus, targeting BACE2 may represent a potential therapeutic strategy for the treatment of this disease. Here, we aimed to investigate the effects of BACE2 suppression on glucose homeostasis in a model of diet-induced obesity.

**Methods:**

BACE2 knock-out (BKO) and wild-type (WT) mice were fed with a high-fat diet (HFD) for 2 or 16 weeks. Body weight, food intake, respiratory exchange ratio, locomotor activity, and energy expenditure were determined. Glucose homeostasis was evaluated by glucose and insulin tolerance tests. β-cell proliferation was assessed by Ki67-positive nuclei, and β-cell function was determined by measuring glucose-stimulated insulin secretion. Leptin sensitivity was evaluated by quantifying food intake and body weight after an intraperitoneal leptin injection. Neuropeptide gene expression and insulin signaling in the mediobasal hypothalamus were determined by qPCR and Akt phosphorylation, respectively.

**Results:**

After 16 weeks of HFD feeding, BKO mice exhibited an exacerbated body weight gain and hyperphagia, in comparison to WT littermates. Glucose tolerance was similar in both groups, whereas HFD-induced hyperinsulinemia, insulin resistance, and β-cell expansion were more pronounced in BKO mice. In turn, leptin-induced food intake inhibition and hypothalamic insulin signaling were impaired in BKO mice, regardless of the diet, in accordance with deregulation of the expression of hypothalamic neuropeptide genes. Importantly, BKO mice already showed increased β-cell proliferation and glucose-stimulated insulin secretion with respect to WT littermates after two weeks of HFD feeding, before the onset of obesity.

**Conclusions:**

Collectively, these results reveal that BACE2 suppression in an obesogenic setting leads to exacerbated body weight gain, hyperinsulinemia, and insulin resistance. Thus, we conclude that inhibition of BACE2 may aggravate the adverse metabolic effects associated with obesity.

## Highlights

• BACE2 KO mice fed HFD (BKO-HFD) exhibit hyperphagia and exacerbated body weight gain.
• β-cell expansion, hyperinsulinemia, and insulin resistance are enhanced in BKO-HFD mice.
• Leptin function and hypothalamic insulin signaling are impaired in BKO mice.
• Expression of hypothalamic neuropeptide genes is deregulated in BKO-HFD mice.
• BKO-HFD mice display early hyperinsulinemia and β-cell proliferation.

## Graphical abstract

**Figure:**
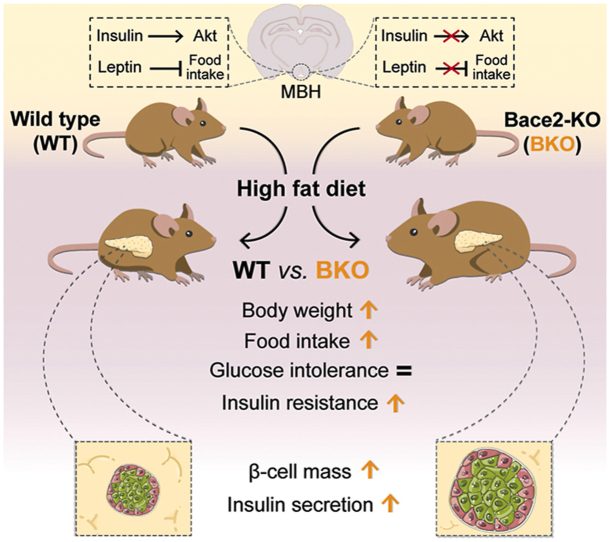

## 1 Introduction

Type 2 diabetes (T2D) is a long-term condition closely linked to the upsurge of obesity [1]. The pathophysiology connecting obesity and T2D is attributed to insulin resistance [2]. Obesity induces insulin resistance affecting peripheral tissues, which results in a compensatory increase of pancreatic insulin secretion. Failure of β-cells to compensate insulin resistance will ultimately lead to T2D. Therefore, strategies targeting β-cell function and expansion can be essential for reestablishing glucose homeostasis in T2D associated with obesity.

The pathophysiology of T2D is complex and, in addition to β-cell function, the control of glucose and energy homeostasis involves the action of several hormones in peripheral tissues such as the liver, skeletal muscle, and adipose tissue, as well as in regulatory regions of the brain. In this regard, leptin is an adipocyte-derived hormone that communicates fuel availability to the central nervous system by suppressing appetite and increasing energy expenditure [3]. Together with insulin, both hormones exert their effects on body weight and fuel metabolism through their actions in specific neuronal populations within the arcuate nucleus of the hypothalamus [4]. Early after the administration of a high-fat diet (HFD), the sustained increase of caloric intake induces hyperphagia and glucose intolerance [5], which are followed by a compensatory increase in leptin and insulin secretion prior to the onset of obesity and diabetes [6,7].

BACE2 (β-site APP-cleaving enzyme 2) is a type I transmembrane protease [8] that has been involved in the control of glucose metabolism [9,10]. Its close homolog BACE1 (β-site APP-cleaving enzyme 1) is highly expressed in the brain and responsible for initiating the amyloid cascade in Alzheimer's disease (AD) [11,12]. BACE2 transcripts have been reported to be expressed at very low levels in different brain regions [10,13,14]. Nevertheless, BACE2 is expressed in discrete subsets of neurons and glia throughout the adult mouse brain and rat brain regions [13,15], although its contribution to the pathogenesis of AD remains controversial [16]. BACE2 is expressed at low levels in most peripheral tissues. Importantly, the highest relative expression of BACE2 has been found in pancreatic islets [10,14], where it is expressed solely in the insulin-producing pancreatic β-cells in both humans and mice [10,17]. We previously demonstrated that inhibition of the β-secretase enzymatic function of BACE2 in pancreatic β-cells affects the intracellular trafficking of the insulin receptor as well as insulin gene expression and content, suggesting that this protease plays an important role maintaining β-cell function [18]. In the same line, the genetic inhibition of BACE2 improves the deficient secretory response of insulin in rat pancreatic β-cell line INS1E transfected with human islet amyloid polypeptide (hIAPP) [14]. Moreover, our group demonstrated that in a mouse model of β-cell dysfunction induced by the overexpression of hIAPP in β-cells, BACE2 suppression reverts glucose intolerance and improves β-cell survival [9]. Of interest, BACE2 was identified as the sheddase of the pro-proliferative plasma membrane protein TMEM27 in murine and human β-cells [10]. Accordingly, insulin-resistant mice treated with a BACE2 inhibitor displayed augmented β-cell mass and improved control of glucose homeostasis due to increased insulin levels [10]. Collectively, these studies suggest that BACE2 inhibition may have a therapeutic potential for T2D treatment.

Pharmacological inhibition of BACE1 is being intensively pursued as a therapeutic approach to treat patients with AD [19]. Although significant progress has been made in the last years, the majority of current BACE1 inhibitors lack selectivity and can also target BACE2 [20,21]. Interestingly, it has been reported that BACE1 suppression decreases body weight, protects against diet-induced obesity and enhances insulin sensitivity in mice [22,23]. The beneficial effects that BACE2 inhibition exerts on β-cell mass and function in different models of T2D suggests that selective BACE2 inhibitors may be potentially a new path for the treatment of this disease [24]. However, the effects of BACE2 inhibition on glucose metabolism under an obesogenic regime has not yet been explored. This prompted us to explore the impact of BACE2 suppression on the regulation of glucose and energy homeostasis in a model of diet-induced obesity. We found that BACE2 knock-out (BKO) mice fed with a HFD presented an exacerbated body weight gain, hyperphagia, hyperinsulinemia, and insulin resistance compared to their wild-type (WT) littermates, indicating that BACE2 suppression aggravates the adverse metabolic effects induced by obesogenic diets.

## 2 Materials and methods

### 2.1 Animals

Bace2^ΔE6^ mice were purchased from The Jackson Laboratory and continued in the B6; 129P2 background [25]. Bace2^ΔE6^ mice were crossed to obtain WT and Bace2^ΔE6/ΔE6^ (BKO) male mice. Mouse genotyping was performed by PCR amplification of ear DNA samples. Mice at 8 weeks of age were individually caged and fed *ad libitum* either with pre-weight chow diet (CD, A04 type, Safe diets, Augy, France) or HFD (45% kcal derived from fat; Research Diets, New Brunswick, NJ, USA) for 2 or 16 weeks. Food intake and animal weight were controlled weekly. Epididymal and subcutaneous inguinal white adipose depots, gastrocnemius muscle and liver were dissected and weighed. Protocols were approved by the Animal Ethics Committee of the Universitat de Barcelona, and the Principles of Laboratory Animal Care were followed.

### 2.2 Indirect calorimetry

Indirect calorimetry was carried out using a 16-chamber TSE Phenomaster monitoring system (TSE Systems GmbH, Bad Homburg, Germany). Individually caged mice were placed into the measuring room one week before the onset of the experiment for acclimation. Mice were fed with CD or HFD as indicated. In the HFD group, the time of indirect calorimetry experiments coincided with the sixteenth week of the diet period. Determination of different parameters was carried out over a period of 48–72 h. Oxygen consumption and CO_2_ production were directly measured. From these data, respiratory exchange ratio (RER) and energy expenditure (EE) were calculated as follows: RER = V_CO2_/V_O2_; EE = (3.185 + 1.232 × RER) x V_O2_.

### 2.3 Locomotor activity

Locomotor activity was simultaneously monitored together with calorimetry parameters. Ambulatory movement in their cages was continuously registered every hour over a period of 48–72 h using an infrared photocell beam grid and is represented as the averaged of the total number of beam breaks in the x-and y-axis during the light and dark periods.

### 2.4 Histochemistry

The left liver lobe was fixed in 4% paraformaldehyde overnight at 4 °C, then was transferred to 30% sucrose in phosphate-buffered saline (PBS) for 24 h at 4 °C and embedded in tissue freezing medium (Leica, Wetzlar, Germany). We obtained 8-μm-thick sections with a CM1860 cryostat (Leica) and applied them to poly-lysine coated slides. Liver sections were stained with Oil red O as described elsewhere [26]. Images were taken with Nikon Eclipse E600 fluorescence microscope and collected with Olympus CellˆD software v3.4.

### 2.5 Glucose and insulin tolerance tests

For intraperitoneal glucose tolerance tests (GTTs), mice were fasted for 12 h and were intraperitoneally injected with d-glucose (2 g/kg body weight). For intraperitoneal Insulin Tolerance Tests (ITTs), mice fasted for 5 h were intraperitoneally injected with 0.4 IU/kg insulin (Humulin-R, Eli Lilly, Indianapolis, IN, USA). Blood glucose levels were measured via tail vein using an automatic glucometer (Nova Pro) at different time points after glucose or insulin injection.

### 2.6 Insulin and leptin determinations

Blood samples were collected from the tail vein of mice fasted for 12 h. Blood samples were also collected 15 min after glucose injection in GTTs. Plasma insulin and leptin levels were measured with ELISA kits according to manufacturer's guidelines (Crystal Chem, Downers Grove, IL, USA).

### 2.7 Glucose-stimulated insulin secretion (GSIS)

Pancreatic islets were isolated from WT and BKO males following the dietary intervention by collagenase digestion and handpicked after a density gradient using Histopaque (Sigma–Aldrich, St Louis, MO, USA), as previously described [27]. The same day of isolation, islets were preincubated with Krebs–Ringer bicarbonate HEPES buffer solution (115 mmol/L NaCl, 24 mmol/L NaHCO_3_, 5 mmol/L KCl, 1 mmol/LMgCl_2_·6H_2_O, 1 mmol/L CaCl_2_·2H_2_O, 20 mmol/L HEPES, and 0.5% BSA, pH 7.4) containing 2.8 mmol/L glucose for 30 min at 37 °C. Eight islets per assay were then incubated with either 2.8 mmol/L glucose or 16.7 mmol/L glucose. After 1 h, supernatants were collected and cellular insulin contents were recovered in acid-ethanol solution. Insulin concentration was determined by Insulin Mouse ELISA (Crystal Chem).

### 2.8 Leptin sensitivity test

Mice were individually housed and acclimatized by handling and sham injections for a week. Ten-week-old WT and BKO mice on HFD for two weeks were intraperitoneally injected with either vehicle or 5.5 mg/kg of recombinant mouse leptin (R&D Systems, Minneapolis, MN, USA) 1 h before lights-out. Food intake and body weights were recorded every 12 h.

### 2.9 Central insulin signaling test

Mice were fasted for 12 h and anesthetized by an intraperitoneal injection of ketamine and xylazine (100 mg/kg and 10 mg/kg, respectively). We injected 100 μL of a 0.9% saline solution with or without 5 U of regular human insulin (Humulin R, Eli Lilly) into the inferior cava vein. After 10 min, the mediobasal hypothalamus (MBH) was isolated using a coronal brain matrix. Insulin signaling was analyzed by determining phosphorylated and total Akt levels by western blot.

### 2.10 Western blot analysis

MBH was homogenized in RIPA buffer (1% Triton X-100, 1% sodium deoxycolate, 0.1% SDS, 0.15 M NaCl, 0.05 M Tris–HCl, pH 7.2) supplemented with complete mini protease inhibitor cocktail tablets (Roche, Mannheim, Germany). An equal amount of protein (15 μg) was heat-denaturized and separated on 12% SDS acrylamide gels and transferred onto PVDF Immobilon-P membrane (Millipore, Burlington, MA, USA). Subsequently, membranes were blocked with 5% non-fat dry milk in Tris-buffered saline including 0.1% Tween-20 (TBS-T; Sigma–Aldrich) and incubated overnight at 4 °C with the appropriate primary antibody in 5% BSA in TBS-T. Membranes were then incubated with primary antibodies against Akt (#9272) and p-Akt (#9271) (Cell Signaling, Danvers, MA, USA), followed by incubation with horseradish peroxidase (HRP)-conjugated anti-rabbit IgG (Cyvita, Marlborough, MA, USA). Immunoreactive bands were visualized using ECL (Millipore) reagent and image acquisition was performed by using a chemiluminescent CCD imager ImageQuant LAS 4000 (GE Healthcare, Velizy-Villacoublay, France). Phosphorylated and total Akt bands were quantified using ImageJ software version 1.0 (National institutes of Health, Bethesda, MD, USA).

### 2.11 Immunofluorescence and quantitative image analysis

Pancreata from WT and BKO mice fed two weeks on CD and HFD were dissected, fixed in 10% formalin neutral buffered solution and paraffin embedded. Three-micrometer-thick pancreatic sections from 3 different levels (>150 μm apart) for each pancreas were deparaffinized, rehydrated, boiled in citrated buffer (10 mM; pH = 6.0) only in case of Ki67 staining, permeabilized with 1% Triton X-100 (Sigma–Aldrich) in Dulbecco's phosphate buffer saline (PBS; Sigma–Aldrich) and blocked with 5% Donkey serum in PBS. Samples were incubated overnight with polyclonal guinea pig anti-insulin (1:500; Dako, Glostrup, Denmark) and mouse-anti glucagon (1:1000; Sigma–Aldrich) or rabbit anti-Ki67 (1:200; Thermo Fisher Scientific, Waltham, MA, USA). As secondary antibodies, Alexa Fluor 488 anti-guinea pig (1:250; Jackson I.R., Newmarket, UK), 555 anti-mouse (1:250; Invitrogen, Carlsbad, CA, USA) and 455 anti-rabbit (1:400, Dako) were used. Hoechst 33342 (Sigma Aldrich) was used to stain nuclei. Images were taken with Leica TCS SPE confocal microscope and the analysis performed using Fiji (ImageJ) version 2.1 [28]. Total β-cell mass was calculated by multiplying their fractional area per pancreas weight. The percentage of proliferating β-cells was calculated as the percentage (%) of Ki67-positive β-cells with respect to total β-cells.

### 2.12 Gene expression analysis

Total RNA from adipose tissue and mediobasal hypothalamus (MBH) was isolated using Trizol (Sigma–Aldrich) reagent and reverse-transcribed using SuperScript (Invitrogen). qPCR was conducted using SYBR Green (Invitrogen) in ABI Prism 7900 HT Sequence Detection System (Applied Biosystems, Foster City, CA, USA). The primers used were as follows: *Hprt* sense 5′-GGTTAAGCAGTACAGCCCCA-3′, *Hprt1* antisense 5′-TCCAACACTTCGAGAGGTCC-3′; *Leptin* sense 5′-CATCTGCTGGCCTTCTCCAA-3′, *Leptin* antisense 5′-ATCCAGGCTCTCTGGCTTCTG-3′; *Npy* sense 5′-TACTCCGCTCTGCGACACTA-3′, *Npy* antisense 5′-TACTCCGCTCTGCGACACTA-3′; *Agrp* sense 5′-AGCTTTGTCCTCTGAAGCTGT-3′, *Agrp* antisense 5′-AGCTTTGTCCTCTGAAGCTGT-3′; *Pomc* sense 5′-CCTTTCCGCGACAGAGACTA-3′, *Pomc* antisense 5′-AGCGAGAGGTCGAGTTTGC-3′. Expression levels were normalized to the expression of an endogenous house-keeping gene (*Hprt1*).

### 2.13 Statistical analysis

All results are expressed as mean ± standard error of mean (SEM). The D'Agostino & Pearson omnibus normality test was carried out to test whether the variables followed a normal distribution (alpha = 0.05). Statistical significance was determined by a paired two-tailed Student's t-test or two-way ANOVA with Tukey's or Fisher's least significant difference (LSD) *post hoc* analysis as indicated. A value of *P* < 0.05 was considered statistically significant. All figures and statistical analyses were generated using GraphPad Prism 8 (Graphpad Software, La Jolla, CA, USA).

## 3 Results

### 3.1 BKO mice develop exacerbated obesity and hyperphagia with altered energy metabolism after long-term HFD feeding

**Figure 1:**
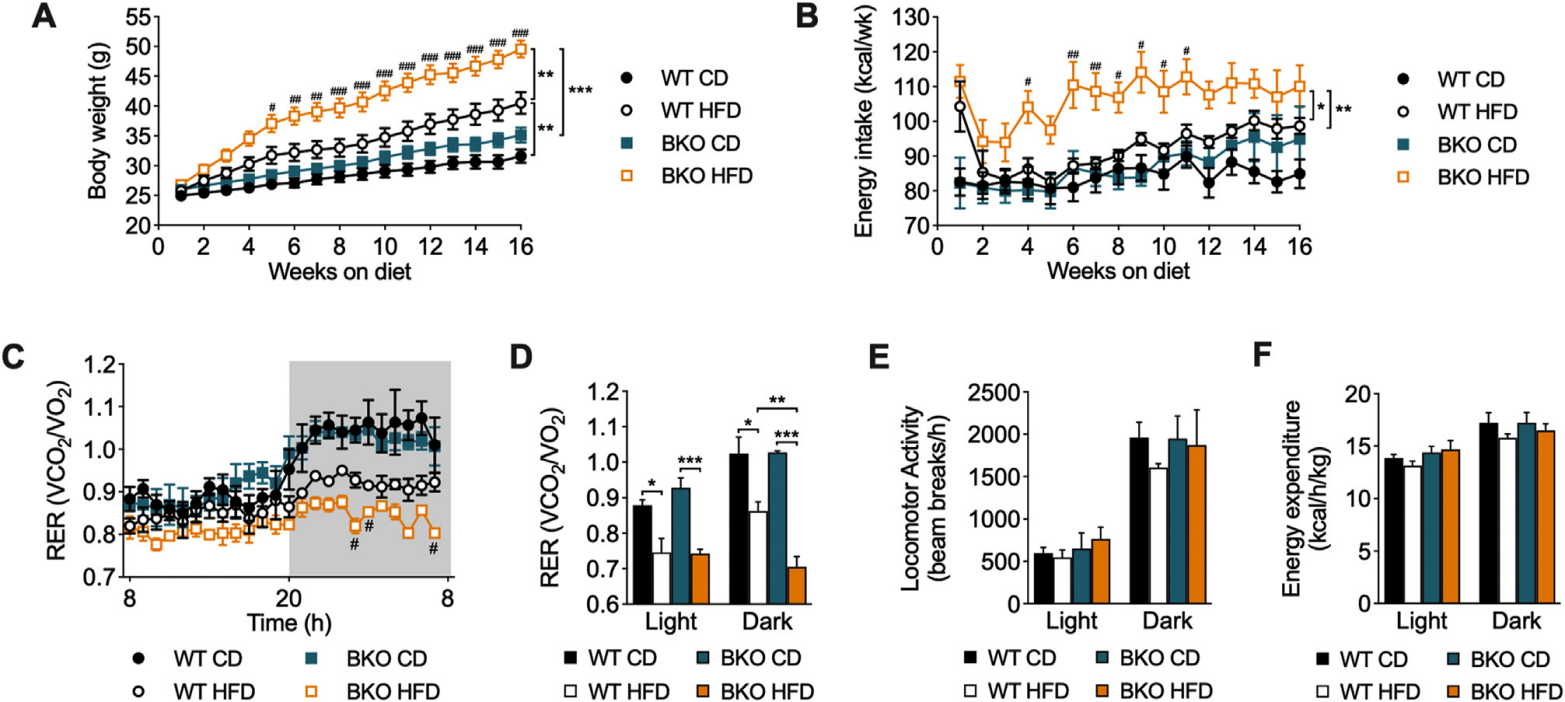
**Body weight, energy intake, respiratory exchange ratio, and energy expenditure of BKO mice after long-term HFD feeding**. (A) Evolution of body weight of WT and BKO mice on chow diet (WT CD, n = 15; BKO CD, n = 17) or high-fat diet (WT HFD, n = 13; BKO HFD, n = 16) and (B) energy intake (n = 5–7 mice/group) during 16 weeks of dietary treatment. (C) Respiratory exchange ratio (RER) corresponding to a complete 24 h cycle (the gray area represents the dark phase) (n = 3–4 mice/group). (D) Average of RER values represented in C. (E) Locomotor activity and (F) energy expenditure measured during the light and dark phases. Data represent the mean ± SEM. Two-way ANOVA multiple comparisons with Tukey's *post hoc* test (A–F). ∗*P* < 0.05, ∗∗*P* < 0.01, ∗∗∗*P* < 0.001; ^#^*P* < 0.05, ^##^*P* < 0.01, ^###^*P* < 0.001 (WT-HFD *vs*. BKO-HFD).

**Supplementary Figure S1:**
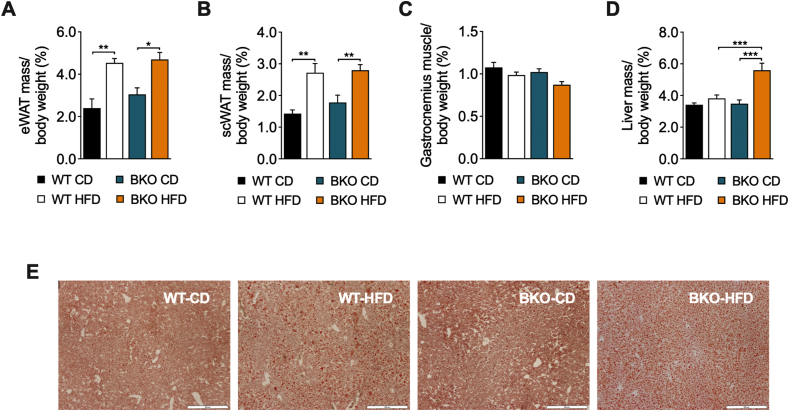
**Weights of adipose depots, gastrocnemius muscle and liver of mice after a long-term HFD feeding.** Percentage of the weights of (A) epididymal and (B) inguinal subcutaneous white adipose tissues (eWAT and scWAT, respectively), (C) gastrocnemius muscle, and (D) liver respect to total body weight of WT and BKO mice on chow diet (WT CD, n = 6–13; BKO CD, n = 6–15) or high-fat diet (WT HFD, n = 5–14; BKO HFD, n = 4–13) for 16 weeks. (E) Representative Oil Red O staining of liver sections (n = 5–6 mice/group) from WT and BKO mice fed either CD or HFD for 16 weeks. Scale bar, 500 μm. Data represent the mean ± SEM. Two-way ANOVA multiple comparisons with Tukey's *post hoc* test (A-D). ∗*P* < 0.05, ∗∗*P* < 0.01, ∗∗∗*P* < 0.001.

To study the implication of BACE2 in the regulation of energy homeostasis under obesogenic conditions, we monitored the body weight and food intake of WT and BKO mice fed either with chow diet (CD) or HFD for 16 weeks. Both HFD groups gained more weight than the groups on CD, but remarkably, BKO-HFD mice gained more weight than WT-HFD mice (Figure 1A). Thus, HFD administration induced a body weight gain of 92.5 ± 4.3% in BKO mice as compared to only 66.2 ± 6.9% in WT mice (Figure 1A). Both HFD groups displayed a similar increase in adiposity, measured as the percentage of the weight of epididymal white adipose tissue and subcutaneous adipose tissue with respect to body weight. Liver weight was significantly higher in BKO-HFD mice than in the other groups, which was accompanied by enhanced accumulation of hepatic lipid droplets as demonstrated by Oil Red O staining of liver sections (Supplementary Figure S1). In parallel, we also measured the energy intake (EI) on a weekly basis. Importantly, BKO-HFD mice exhibited increased hyperphagia compared to WT-HFD mice, which presented similar values to mice under CD feeding (Figure 1B). This increase in food intake was observed shortly after initiating the dietary treatment and prior to a significant weight gain, suggesting an early deregulation of food intake in BKO mice when challenged with a HFD.

Subsequently, we performed indirect calorimetry to gain insights into the metabolic status of mice. Under CD conditions, both groups presented similar oscillations in RER (Figure 1C,D). HFD groups showed a lower RER than CD mice with a reduced oscillatory pattern, consistent with a preference for lipid oxidation as a source of fuel. Remarkably, RER was significantly lower in BKO-HFD mice compared to WT-HFD mice during the active dark cycle. These results show a metabolic profile that is consistent with the enhanced body weight observed in BKO-HFD mice. Importantly, no significant differences were observed in locomotor activity (Figure 1E) and energy expenditure normalized to body weight (Figure 1F) during the dark and light periods. Taken together, these results indicate that the increased body weight observed in BKO-HFD mice is mainly due to enhanced energy intake rather than a decrease in activity or energy expenditure.

### 3.2 BKO mice exhibit enhanced insulin secretion under long-term HFD feeding

**Figure 2:**
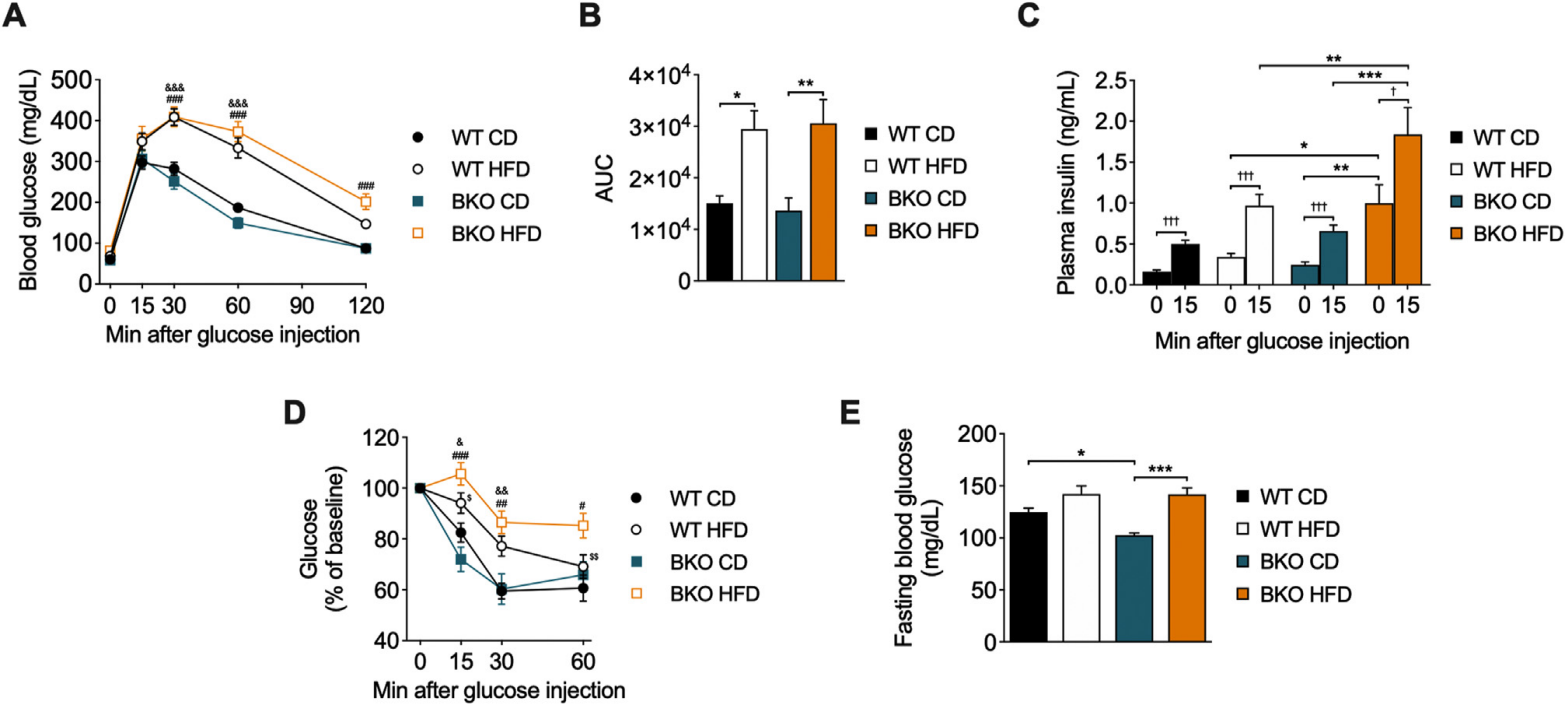
**Glucose homeostasis and insulin secretion in BKO mice after long-term HFD feeding**. (A) Intraperitoneal glucose tolerance test (GTT) (n = 11–17 mice/group), (B) Area under the curve (AUC) from the GTT, (C) plasma insulin levels before and 15 min after glucose administration (n = 11–18 mice/group), (D) intraperitoneal insulin tolerance test (ITT) (n = 12–15 mice/group) and (E) fasting blood glucose levels (n = 15–19 mice/group) in WT and BKO mice fed either CD or HFD for 16 weeks. Data represent the mean ± SEM. Indicated comparisons were made using two-way ANOVA followed by Tukey's (A, B, C and E) or Fisher's LSD (D) *post hoc* tests. ^&^*P* < 0.05, ^&&^*P* < 0.01, ^&&&^*P* < 0.001 (WT-CD *vs.* WT-HFD); ^#^*P* < 0.05, ^##^*P* < 0.01, ^###^*P* < 0.001 (BKO-CD *vs.* BKO-HFD); ^$^*P* < 0.05, ^$$^*P* < 0.01 (WT-HFD *vs*. BKO-HFD); ∗*P* < 0.05, ∗∗*P* < 0.01, ∗∗∗*P* < 0.001. ^†^*P* < 0.05, ^†††^*P* < 0.001, using Student's paired t-test (C).

To determine the effects of BACE2 suppression on whole-body glucose homeostasis, mice were challenged with an intraperitoneal glucose tolerance test (GTT). As expected, long-term HFD administration resulted in a decrease in glucose tolerance when compared with CD groups (Figure 2A,B). Interestingly, BKO-HFD mice displayed a similar impairment in glucose tolerance compared to WT-HFD mice, despite the higher weight gain observed in BKO-HFD mice. Plasma insulin levels were determined at basal conditions and 15 min after glucose challenge. Remarkably, basal insulin levels were much higher in BKO-HFD in comparison to that of the other groups (Figure 2C). Glucose administration induced insulin secretion in all groups, although it was more pronounced in WT-HFD mice and especially in BKO-HFD mice. These results revealed an exacerbated hyperinsulinemia in BKO-HFD mice at fasting conditions and in response to glucose.

We subsequently evaluated insulin sensitivity by an ITT. Insulin sensitivity was decreased in both HFD-fed mice groups compared with their respective CD groups (Figure 2D). Despite similar levels of glucose intolerance between the two HFD groups, BKO-HFD mice were more insulin-resistant than the WT-HFD mice, in line with the increased insulin levels. Of note, BKO-CD mice showed lower levels of fasting glucose than their WT-CD littermates (Figure 2E), with the same insulin sensitivity, which could be attributable to the slightly increased basal insulin levels in BKO-CD mice (0.25 ± 0.04 ng/mL) as compared to WT-CD mice (0.16 ± 0.02 ng/mL).

### 3.3 Pancreatic islets from BKO mice under HFD feeding display an exacerbated insulin secretion and β-cell proliferation

**Figure 3:**
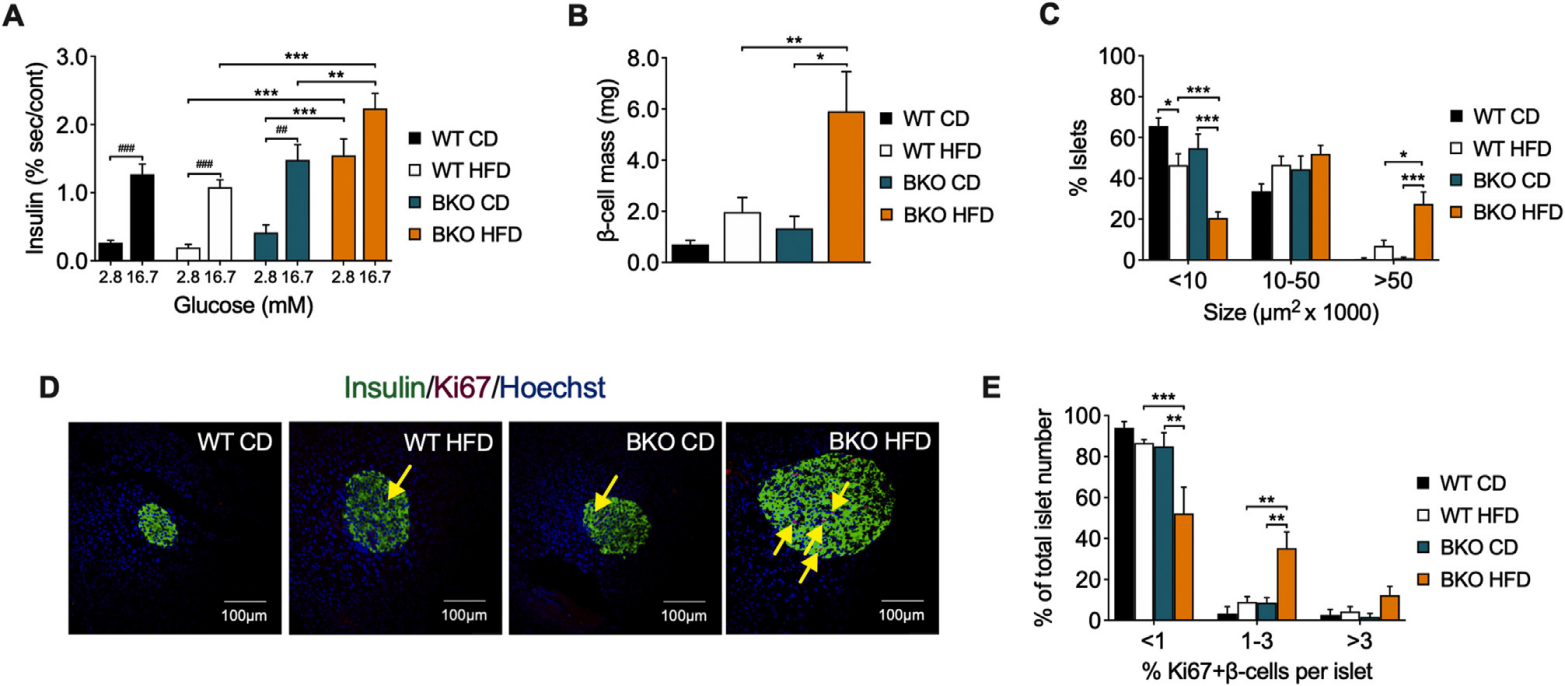
**Enhanced insulin secretion and β-cell mass and proliferation in BKO mice after long-term HFD feeding**. (A) *Ex vivo* glucose-stimulated insulin secretion at 2.8 mM and 16.7 mM of glucose, represented as % of insulin secretion respect to insulin content of islets from WT and BKO mice fed either CD or HFD for 16 weeks (n = 3 mice/group). (B) Quantification of β-cell mass and (C) profile of islet size distribution in WT and BKO mice fed either CD or HFD for 16 weeks (n = 5–7 replicates/group). (D) Representative images of islets stained for insulin (green), Ki67 (red) and nuclei with Hoechst (blue). The yellow arrows point to Ki67 positive nuclei in insulin-positive cells. Scale bar, 100 μm. (E) Quantification of β-cell proliferation in pancreas from WT and BKO mice fed either CD or HFD for 16 weeks (n = 3 mice/group). Three sections per animal were analyzed. Data represent the mean ± SEM. ∗*P* < 0.05, ∗∗*P* < 0.01, ∗∗∗*P* < 0.001, using two-way ANOVA followed by Tukey's *post hoc* test (A, B, C and E). ^##^*P* < 0.01, ^###^*P* < 0.001, using Student's paired t-test (A).

To assess the impact of BACE2 suppression on the secretory profile of the pancreatic β-cells, we isolated pancreatic islets and performed glucose-stimulated insulin secretion (GSIS) tests. Islets from all groups had a similar secretory response to high glucose except those from BKO-HFD mice, which displayed a higher basal insulin secretion and a relatively reduced response to high glucose, even though the total amount of insulin released was higher than that of any other group (Figure 3A).

Morphometric analysis of pancreas sections revealed that the β-cell mass and the frequency of large islets were increased in BKO-HFD mice as compared to BKO-CD and WT-HFD mice (Figure 3B,C). In line with these results, the number of proliferating β-cells, identified by double staining of insulin and Ki67, was also increased in the BKO-HFD mice (Figure 3D,E). Thus, the increased plasma insulin levels in BKO mice following 16 weeks of HFD feeding were associated with an enhanced expansion of the β-cell mass.

### 3.4 BKO mice show impaired leptin response and hypothalamic insulin signaling with altered neuropeptide expression

**Figure 4:**
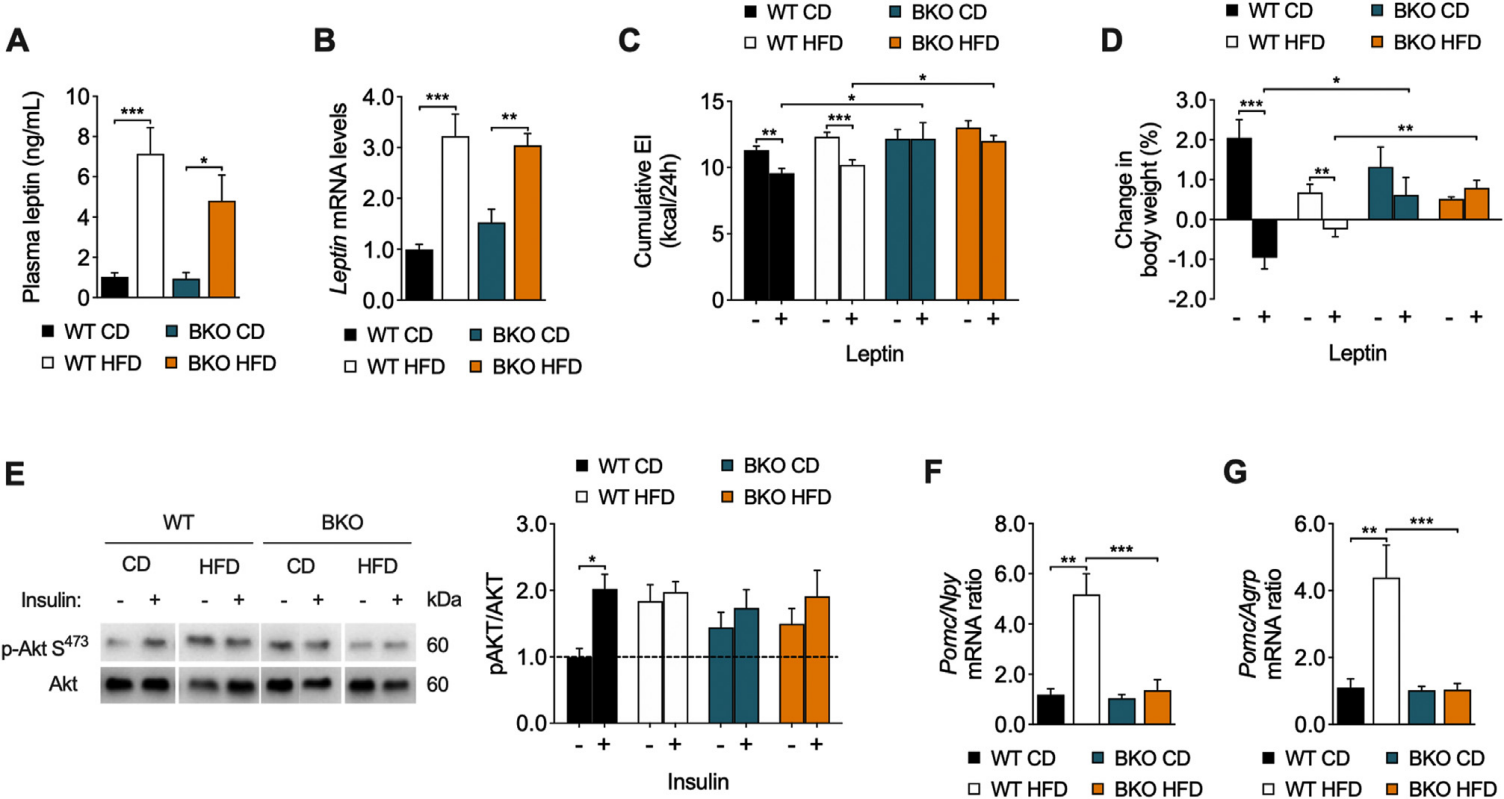
**BKO mice exhibit impaired leptin sensitivity and central insulin signaling**. (A) Circulating leptin plasma levels (n = 5–7/group) and (B) leptin mRNA expression in epididymal white adipose tissue (n = 5–6/group) in WT and BKO mice fed either CD or HFD for 2 weeks. (C) Cumulative energy intake and (D) change in body weight 24 h after an intraperitoneal injection of leptin in WT and BKO mice fed CD or HFD for 2 weeks (n = 5–6 mice/group). (E) Representative western blots for phospho-Akt (p-Akt S^473^) and Akt in the mediobasal hypothalamus from WT and BKO mice fed CD or HFD for 2 weeks after an intraperitoneal injection of insulin. The graphs on the right represent the ratios of p-Akt S^473^ to total Akt quantifications normalized to the basal values of WT mice on CD (n = 6–9/group). (F,G) Ratios between *Pomc*, and *Npy* (F) or *Agrp* (G) mRNA levels in the mediobasal hypothalamus of WT and BKO mice fed CD or HFD for 2 weeks (n = 3–6 mice/group). Data represent the mean ± SEM. ∗*P* < 0.05, ∗∗*P* < 0.01, ∗∗∗*P* < 0.001, using two-way ANOVA followed by Tukey's *post hoc* test (A–G).

Having observed that BKO-HFD mice display increased body weight, hyperphagia, and hyperinsulinemia in comparison to WT-HFD after 16 weeks of HFD feeding, we next sought to study these mice following a short HFD regime. In fact, BKO-HFD presented enhanced energy intake shortly after the start of the dietary treatment (Figure 1B), suggesting that the mechanisms underlying this phenotype would be early events. First, we evaluated circulating levels of leptin after two weeks on HFD. In fasting conditions, the levels of leptin were similarly increased in both HFD-fed mice groups (Figure 4A). Consistently, the mRNA expression of leptin in the epididymal white adipose tissue was also increased in both groups (Figure 4B). These results ruled out the possibility that differences in energy intake may be explained by differences in circulating leptin levels.

Given that energy intake is regulated in part by leptin and insulin at the hypothalamus, we next sought to assess how the action of these hormones was affected in the absence of BACE2. First, a leptin sensitivity test was performed following two weeks of HFD feeding, corresponding to 10 weeks of mice age. As expected, leptin administration led to an anorexigenic effect in WT-CD mice, as shown by a reduction in food intake (Figure 4C) and body weight (Figure 4D), and this response was attenuated in WT-HFD mice. Remarkably, BKO mice were totally unresponsive to leptin, regardless of the dietary treatment (Figure 4C,D). Because leptin synergistically acts with insulin to induce its anorexigenic actions, and considering that hypothalamic insulin resistance has been show to underlie deregulation in food intake [4], we wondered whether insulin signaling in the hypothalamus may be affected after a short-term HFD feeding. To test this, an intravenous bolus of insulin or saline was administered to mice in order to analyze the activation of Akt, a proxy for insulin signaling, in the mediobasal hypothalamus (MBH). As expected, insulin administration resulted in Akt activation in WT-CD mice (Figure 4E). Conversely, the basal phosphorylation of Akt was already increased in the WT-HFD mice and in both BKO groups and hence insulin was unable to further activate Akt. Collectively, these results revealed an impaired anorexigenic response to leptin and insulin signaling in the MBH of BKO mice, even when fed on CD, which may trigger the increase in food intake and body weight observed in BKO mice fed on HFD.

**Supplementary Figure S2:**
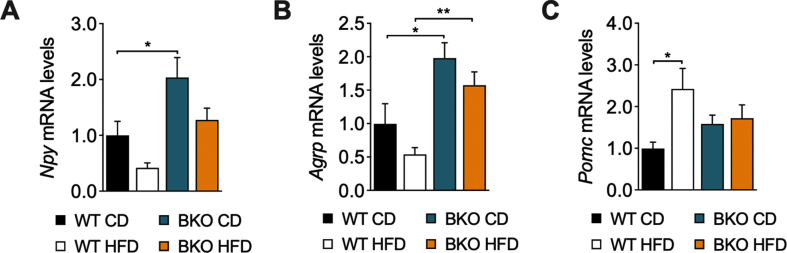
**Expression levels of genes encoding neuropeptides in mice after a short-term HFD feeding**. mRNA expression levels of (A) *Npy*, (B) *Agrp* and (C) *Pomc* in the mediobasal hypothalamus of WT and BKO mice fed CD or HFD for 2 weeks (n = 3–6 mice/group). Gene expression data were normalized against *Hprt*, and are shown relative to WT-CD, which were set arbitrary to 1. Data represent the mean ± SEM. ∗*P* < 0.05, ∗∗*P* < 0.01, ∗∗∗*P* < 0.001, using two-way ANOVA followed by Tukey's *post hoc* test (A-C).

Next, we assessed the expression of appetite-regulating neuropeptide genes in the MBH. For this, we determined the expression of the genes encoding the orexigenic peptides, neuropeptide Y (*Npy*) and agouti-related protein (*Agrp*), and the anorexigenic peptide proopiomelanocortin (*Pomc*) after an overnight fasting. The *Npy* and *Agrp* expression was significantly increased in the BKO mice, whereas *Pomc* levels were increased in WT-HFD mice in comparison to WT-CD mice; however, this transition between diets was not observed in BKO mice (Supplementary Figure S2). In order to estimate the anorexigenic/orexigenic tone, the ratios between *Pomc* mRNA, and *Npy* or *Agrp* mRNAs were calculated, revealing that the anorexigenic/orexigenic balance was potently enhanced in WT mice after two weeks on HFD (Figure 4F,G). Conversely, the ratios between anorexigenic and orexigenic peptides did not show this transition when BACE2-deficient mice were fed on HFD, suggesting imbalanced orexigenic and anorexigenic outputs in the hypothalamus, in agreement with the enhanced food intake observed in BKO-HFD mice.

### 3.5 BKO mice show early hyperinsulinemia and insulin resistance following a short-term HFD regime

**Figure 5:**
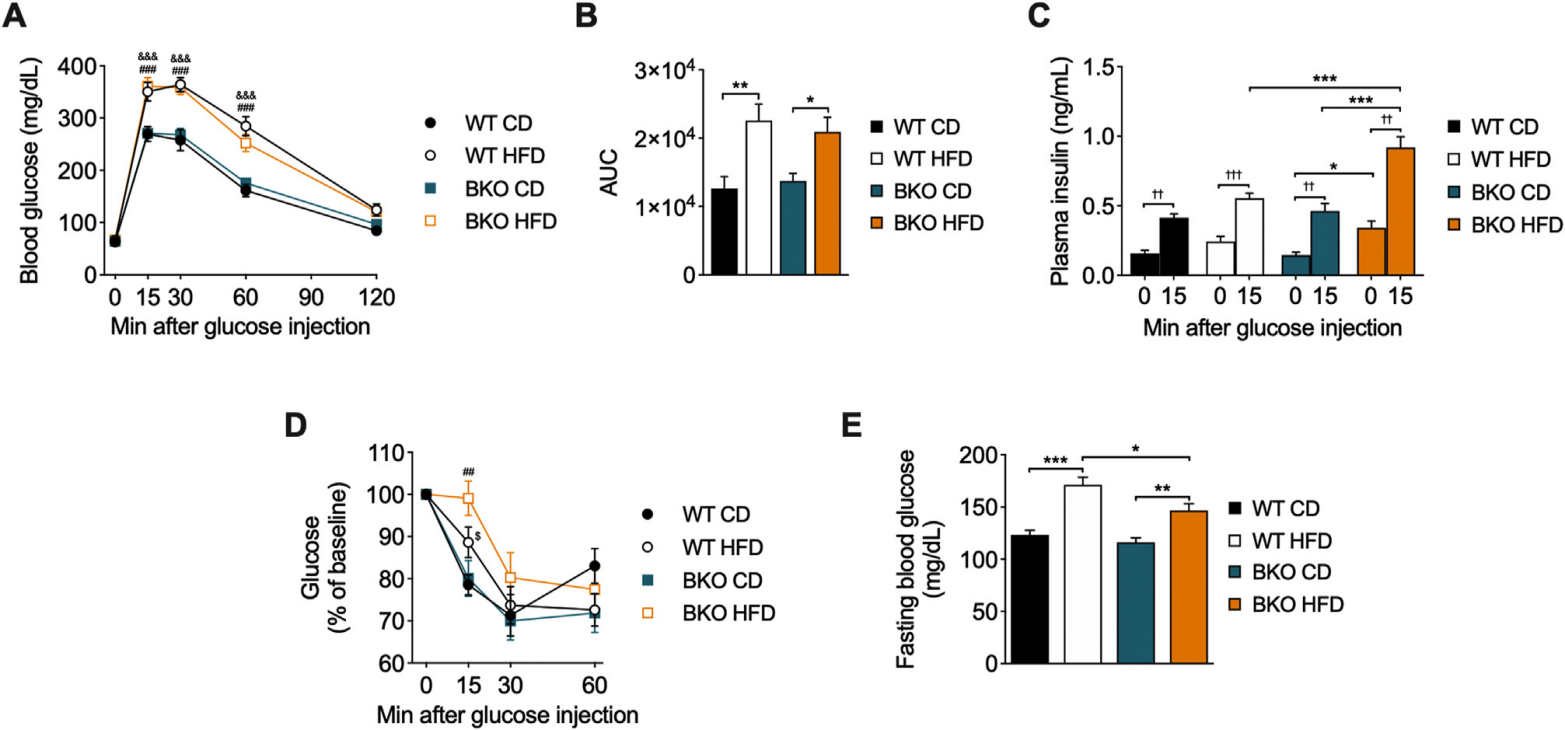
**Glucose homeostasis and insulin secretion in BKO mice after short-term HFD feeding**. (A) Intraperitoneal glucose tolerance test (GTT) (n = 9–11 mice/group), (B) area under the curve (AUC) from the GTT, (C) plasma insulin levels before and 15 min after glucose administration (n = 8–12 mice/group), (D) intraperitoneal insulin tolerance test (ITT) (n = 6–7 mice/group) and (E) fasting blood glucose levels (n = 20 mice/group) in WT and BKO mice fed either CD or HFD for 2 weeks. Data represent the mean ± SEM. ^&&&^*P* < 0.001 (WT-CD *vs.* WT-HFD); ^##^*P* < 0.01, ^###^*P* < 0.001 (BKO-CD *vs*. BKO-HFD); ^$^*P* < 0.05 (WT-HFD *vs.* BKO-HFD); ∗*P* < 0.05, ∗∗*P* < 0.01, ∗∗∗*P* < 0.001, using two-way ANOVA followed by Tukey's (A, B, C and E) and Fisher's LSD (D) *post hoc* tests. ^††^*P* < 0.01, ^†††^*P* < 0.001, using Student's paired t-test (C).

Due to the impairment in leptin and insulin responses in the hypothalamus of BKO mice and the deregulation of appetite regulatory genes shortly after the administration of HFD, we next assessed glucose homeostasis at the same time point. After two weeks of dietary treatment, glucose tolerance was already decreased in both HFD-fed mice groups compared to CD groups (Figure 5A,B). As observed after 16 weeks of HFD feeding, both WT-HFD and BKO-HFD mice exhibited the same level of glucose intolerance. Fasting insulinemia in BKO-HFD mice (0.34 ± 0.05 ng/mL) was slightly increased relative to WT-HFD mice (0.24 ± 0.04 ng/mL) and significantly higher compared with BKO-CD (0.14 ± 0.02 ng/mL) mice (Figure 5C). After glucose administration, plasma insulin levels in the BKO-HFD mice were significantly increased compared with WT-HFD mice and the BKO-CD mice, revealing an early higher secretory response in BKO-HFD mice. The ITT revealed that the HFD groups showed less sensitivity to insulin, implying that BKO-HFD mice are more insulin resistant than their WT HFD littermates (Figure 5D). HFD groups presented higher fasting glycemia than their respective CD groups (Figure 5E). These data indicate that early at two weeks of HFD, the BKO mice display an exacerbated insulin secretion both at fasting and after glucose load and insulin resistance.

### 3.6 Pancreatic islets from BKO mice present a more pronounced β-cell proliferation and insulin secretion following a short HFD feeding

**Figure 6:**
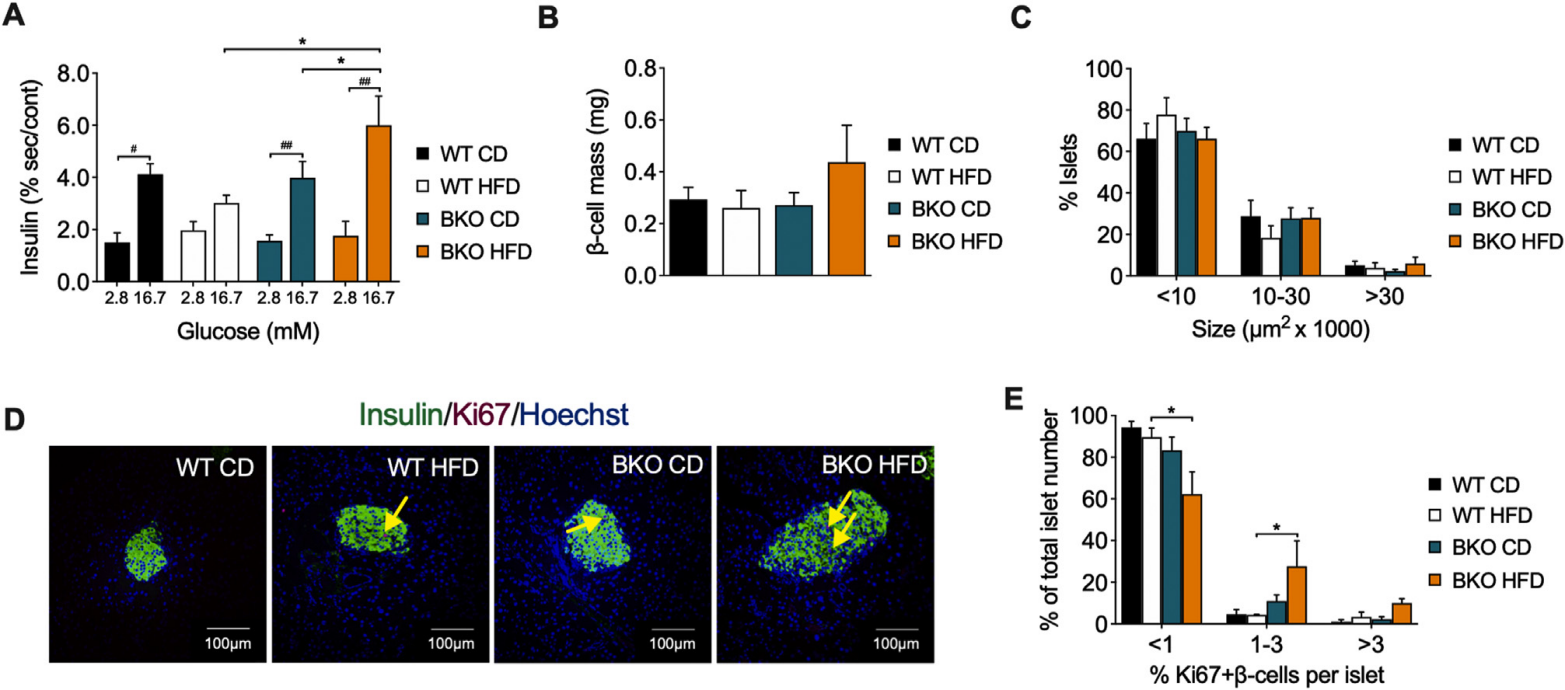
**Enhanced insulin secretion and proliferation in pancreatic islets from BKO mice after short-term HFD feeding**. (A) *Ex vivo* glucose-stimulated insulin secretion at 2.8 mM and 16.7 mM of glucose, represented as % of insulin secretion respect to insulin content of islets from WT and BKO mice fed either CD or HFD for 2 weeks (n = 3 mice/group). (B) Quantification of β-cell mass and (C) profile of islet size distribution in WT and BKO mice fed either CD or HFD for 2 weeks (n = 4–6 replicates/group). (D) Representative images of islets stained for insulin (green), Ki67 (red) and nuclei with Hoechst (blue). The yellow arrows point to Ki-67 positive nuclei in insulin-positive cells. Scale bar, 100 μm. (E) Quantification of β-cell proliferation in pancreas from WT and BKO mice fed either CD or HFD for 2 weeks (n = 3 mice/group). Three sections per animal were analyzed. Data represent the mean ± SEM. ∗*P* < 0.05, using two-way ANOVA followed by Tukey's *post hoc* test (A, B, C and E). ^#^*P* < 0.05, ^##^*P* < 0.01, using Student's paired t-test (A).

Once characterized the metabolic state of mice after two weeks on HFD, we performed GSIS assays in isolated pancreatic islets to further determine the secretory capacity of β-cells. Islets from all groups similarly responded to high glucose, except the WT-HFD mice, whose insulin secretory response was attenuated (Figure 6A). Remarkably, islets from BKO-HFD mice showed an increased insulin secretory response to high glucose when compared with WT-HFD and BKO-CD islets (Figure 6A). Morphometric analyses of pancreas sections revealed no significant differences in β-cell mass and islet size frequency between the experimental groups, although there was already a trend toward a higher β-cell mass in BKO-HFD mice (Figure 6B,C). Indeed, the frequency of islets with a higher percentage of Ki67-positive β-cells was increased in BKO-HFD mice (Figure 6D,E). All together, these results revealed that suppression of BACE2 function leads to an enhanced β-cell secretory capacity and proliferation early after HFD administration.

## 4 Discussion

We previously demonstrated that the absence of BACE2 improves glucose tolerance and insulin secretion in BACE2 KO mice crossed with Tg-hIAPP mice, which display insulin secretory defects due to the aggregation of hIAPP [9]. Here, we aimed to explore whether the absence of BACE2 could affect glucose and energy homeostasis in an obesogenic context. To achieve this objective, WT and BKO mice were challenged with HFD, in order to induce metabolic changes similar to early stages of T2D in humans. This strategy provides a useful tool to study potential therapies for T2D associated with obesity [29,30]. Unexpectedly, we found that, after 16 weeks of HFD feeding, BKO mice gained more weight than the WT mice. The exacerbated increase in body weight was due to a higher energy intake rather than a decrease in activity or energy expenditure. Furthermore, the BKO-HFD mice exhibited a lower RER than the WT-HFD mice, reflecting an increased preference for lipid oxidation as a source of fuel energy.

In parallel, we analyzed the influence of the obesogenic diet on glucose homeostasis in BKO mice. Importantly, glucose intolerance was similar in both HFD groups despite the more accentuated obese phenotype of BKO mice in relation to WT littermates. An explanation for this protection against an expected aggravation of glucose tolerance is the fact that BKO-HFD mice exhibited increased circulating insulin levels at fasting conditions and after a glucose challenge. In addition, the insulin secretory response to glucose in isolated pancreatic islets from BKO-HFD mice was increased compared to those from WT-HFD mice. These results are in agreement with our previous studies showing that BACE2 inhibition in β cells results in enhanced glucose-induced insulin secretion [14]. The more pronounced hyperinsulinemia detected in BKO-HFD mice was associated with increased β-cell mass and proliferation. In this regard, previous studies showed that BACE2 inhibition in genetically obese *ob/ob* mice leads to increased insulin secretion and improved glucose tolerance [10]. In the present study, we show that challenging BKO mice with an obesogenic diet resulted in increased plasma insulin levels but also in a more pronounced impairment of insulin resistance already after two weeks of HFD feeding, before the onset of obesity.

We also focused our attention on understanding the increase in energy intake that leads BKO-HFD mice to gain more weight than control littermates. It is well known that insulin acts as a satiety signal in the brain and reduces food intake, similar to the adipose-derived hormone leptin [4]. Indeed, insulin and leptin synergistically act in the arcuate nucleus via PI3K/Akt [31]. Therefore, we wondered whether the disturbed food intake and glucose homeostasis in BKO mice in the long-term HFD regime may be caused by an early impairment in the central nervous system. For this purpose, we analyzed the anorexigenic response to leptin and the hypothalamic insulin signaling after two weeks of HFD, before the onset of obesity. Surprisingly, our results showed that food intake and body weight reduction in response to leptin administration were attenuated in BKO mice as compared to WT animals, regardless of the diet. Furthermore, we observed that insulin administration was not able to activate Akt in the mediobasal hypothalamus (MBH) in both HFD groups but also in BKO mice fed with CD.

Leptin and insulin mainly exert their anorectic effect in the arcuate nucleus via inhibition of orexigenic NPY/AgRP neurons and activation of anorexigenic POMC neurons resulting in reduced food intake and increased energy expenditure [4]. In this regard, HFD attenuated the expression of the orexigenic *Npy* and *Agrp* genes and increased the expression of the anorexigenic gene *Pomc*, most likely reflecting an attempt to moderate energy intake. Conversely, these transitions favoring an anorexigenic tone were not observed when BKO mice were fed a HFD. Importantly, these effects were already observed after two weeks of HFD treatment, demonstrating an early impairment of the regulation of the expression of hypothalamic neuropeptides controlling food intake, which is in line with the hyperphagia observed in BKO-HFD mice shortly after initiating the diet intervention. Collectively, these results revealed impaired central insulin and leptin responses in the BKO mice under chow diet, suggesting that BACE2 may play a role in hypothalamic areas controlling food intake. It should be noted that although BACE2 expression is highest in pancreatic islets, and at much lower levels in some peripheral tissues, this secretase is expressed in discrete subsets of neurons and glia throughout the adult brain, including the ventromedial hypothalamic nucleus [13,15], and therefore a potential role of BACE2 in the brain requires further investigation. In this regard, it has been shown that proinflammatory TNF induces an increase in BACE2-mediated shedding of vascular cell adhesion molecule 1 in the cerebrospinal fluid from adult mice, illustrating that BACE2 inhibition might become apparent under physiopathological conditions [15]. Furthermore, the effects of BACE2 suppression on hypothalamic signaling and gene regulation also open the possibility that this may impact other regions regulated by hypothalamic outputs. Indeed, the autonomic nervous system modulates the secretory response of pancreatic islets through sympathetic and parasympathetic fibers [33,34], and different hypothalamic areas have been shown to be connected to efferent autonomic fibers innervating pancreatic islets in mice [35]. In the current study, BKO-HFD pancreatic islets *ex vivo* showed a pattern of exacerbated glucose-stimulated insulin secretion similar to that observed *in vivo*. This, together with the increased β-cell mass, suggests that the enhanced insulin secretion in BKO-HFD mice is mainly attributed to a direct effect of BACE2 suppression on pancreatic islets.

Previous studies have demonstrated beneficial effects of BACE1 inhibition on whole-body metabolic homeostasis and neuronal leptin sensitivity under a chronic HFD consumption [22,23], pointing towards distinct roles of BACE1 and BACE2 on metabolism homeostasis. These differences may be explained by the different substrates and tissue expression pattern of each of these secretases. One of the substrates of BACE2 is TMEM27, which is also highly expressed in the pancreatic islets [10]. Nevertheless, other β-cell enriched substrates of BACE2 in β-cells have been identified [32,36]. The molecular mechanisms by which BACE2 deficiency and inhibition promote β-cell function and proliferation are still unresolved, but likely involve the stabilization of TMEM27 as well as other BACE2 substrates yet to be identified that may synergistically contribute to these effects.

Hyperinsulinemia is one of the hallmarks of T2D associated with obesity. In fact, the physiological response to insulin resistance in the early stages of obesity is mainly attributed to an increase in pancreatic β-cell mass and insulin secretion. Nevertheless, increased insulin levels may result in adverse effects on glucose metabolism. On the one hand, insulin induces fat storage in adipose tissue [37], and therefore an excess of insulin may exacerbate fat accumulation in obesogenic contexts. Indeed, mild suppression of insulin secretion has been proven as a strategy for preventing and treating obesity [38,39]. On the other hand, hyperinsulinemia may cause insulin resistance even prior to the onset of obesity [2,40,41]. Indeed, our results show that following a short HFD feeding of two weeks, BKO mice already display enhanced hyperinsulinemia, which may underlie the accentuated insulin resistance in these mice. Moreover, it is also important to stress that hyperinsulinemia in obesity impairs insulin sensitivity in the brain [42,43], leading to deregulation of food intake and eventually body weight gain that may also contribute to the aggravation of the obese phenotype in BKO mice challenged with HFD.

In summary, the present study shows that BACE2 inhibition in mice under a HFD feeding exacerbates body weight gain, hyperphagia, hyperinsulinemia, and insulin resistance. Thus, we conclude that BACE2 suppression may aggravate the adverse metabolic effects associated with an obesogenic diet. These findings should be considered when considering a potential clinical use of non-selective BACE1/BACE2 or selective BACE2 inhibitors for the treatment of AD or T2D, respectively.

## Authors’ contributions

D. D.-C., G. A.-V., C. C., S. P., J. R.-C., A. F.-P., and S. R. contributed to the performance of experiments and data analysis. M. V., M.C., and M. P. contributed to data analysis. D. D-C., A. N., and J.-M. S. wrote the manuscript. A. N. and J.-M. S. designed the study. All authors contributed to the discussion of results, reviewed the manuscript, and approved the final version of this manuscript.
